# Knowledge, attitude and practice related to anthrax among livestock farmers in West Kazakhstan

**DOI:** 10.1002/vms3.1553

**Published:** 2024-07-23

**Authors:** Altyn Kulpiisova, Zukhra Aitpayeva, Assiya Maimatayeva, Lyailya Ussenova, Assel Paritova, Assylbek Zhanabayev, Temirlan Bakishev, Spandiyar Tursunkulov, Tileubek Kitapbay, Aspen Abutalip, Assiya Mussayeva, Yerzhan Ospanov, Urzhan Omarbekova, Bauyrzhan Turalin, Vladislav Sapa, Marat Aisin, Alim Bizhanov, Gulnara Baikadamova, Salbak Chylbak‐ool, Elena Pakhomova, Nurkuisa Rametov, Arman Issimov, Nadezhda Burambayeva

**Affiliations:** ^1^ Department of Veterinary Medicine A. Baitursynov Kostanay Regional University Kostanay Kazakhstan; ^2^ Department of Veterinary Medicine Zhangir Khan West Kazakhstan Agrarian‐Technical University Uralsk Kazakhstan; ^3^ Department of Life Sciences Abai Kazakh National Pedagogical University Almaty Kazakhstan; ^4^ Department of Zootechnology, Genetics andBreeding Toraighyrov University Pavlodar Kazakhstan; ^5^ Department of Veterinary Medicine Saken Seifullin Kazakh Agrotechnical University Nur‐Sultan/Astana Kazakhstan; ^6^ Department of Bacteriology Kazakh Scientific Veterinary Research Institute Almaty Kazakhstan; ^7^ Department of Biology K. Zhubanov Aktobe Regional University Aktobe Kazakhstan; ^8^ Department of Protection in Emergency Situation Russian State Agrarian University – Moscow Timiryazev Agricultural Academy Moscow Russian Federation; ^9^ Department of Geospatial Engineering Satbayev Kazakh National Research Technical University Almaty Kazakhstan

**Keywords:** anthrax, attitude and practice (KAP), Bayterek, knowledge, West Kazakhstan, zoonosis

## Abstract

**Background:**

Anthrax is the most prioritized zoonotic disease in Kazakhstan due to its threatening potential to the public health and agricultural sector. Sporadic anthrax outbreaks are being reported annually among human and livestock populations throughout the country, with the highest frequency occurring in West Kazakhstan.

**Methods:**

A cross‐sectional study was conducted using a survey‐based face‐to‐face interview. From January to May 2022, 489 randomly selected participants were surveyed in 6 districts of the Baiterek province in West Kazakhstan oblast to evaluate the knowledge, attitude and practice (KAP) regarding anthrax among community members. This is the first KAP study conducted relating to outbreaks of anthrax in Kazakhstan.

**Results:**

In this study, most participants (74%) surveyed were males, and 40% of respondents had a secondary level education. Overall, 91% of the community respondents were engaged in agriculture and livestock rearing. Among these community members, cattle rearing was the most common (67%) occupation compared to other livestock species. Additionally, over a 50% of the population studied had no knowledge about the zoonotic nature of the disease, and about 82% and 87% of respondents were unaware of any animal and human anthrax symptoms, respectively. About 70% of the respondents were interested in vaccinating their livestock against anthrax. Individuals aged 45–54 displayed notably higher animal vaccination rates (45%; 95% CI: 38.4–52.0; *p* < 0.025) compared to those aged 25–34 and 65–74. Respondents residing in the Beles district (20%; 95% CI: 17.1–24.7; *p* < 0.005) exhibited a significantly higher level of awareness concerning the fatality of anthrax in contrast to participants from Bolashak. Roughly 61% of respondents held the belief that anthrax is a lethal disease. An overwhelming majority of the survey participants (99%) affirmed their non‐participation in the slaughter of infected animals.

**Conclusion:**

The findings of this study indicate that KAP among community members relating to anthrax is low and requires swift implementation of education programmes in building awareness of anthrax under the One Health approach, especially in anthrax prone regions.

## Graphical abstract

Anthrax poses a significant threat to public health and agriculture in Kazakhstan, particularly in West Kazakhstan, where sporadic outbreaks occur annually. This cross‐sectional study evaluates the knowledge, attitudes and practices (KAP) regarding anthrax among community members in the Bayterek province. The survey, conducted from January to May 2022, involved 489 randomly selected participants from 6 districts. Results reveal limited awareness of anthrax's zoonotic nature and its symptoms among respondents. Despite the high engagement in agriculture and livestock rearing (91%), a significant portion of the population lacks knowledge about anthrax. However, there is a notable interest (70%) in vaccinating livestock against anthrax. Demographic factors such as age and district of residence influence awareness and vaccination rates. The study underscores the urgent need for educational programmes to enhance anthrax awareness and promote preventive measures, especially in high‐risk regions, under the One Health approach.

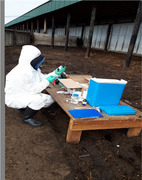

## 1 INTRODUCTION

Anthrax is a highly contagious zoonotic disease that infects humans, livestock and wildlife (Hugh‐Jones & Blackburn, 2009). The causative agent of anthrax is *Bacillus anthracis*, a spore‐forming Gram‐positive bacterium. It is an endemic disease in developing countries and occasionally occurs in developed countries (Dragon & Rennie, 1995; Dutta et al., 2021). The spores of *B. anthracis* can resist harsh ambient conditions, persist in the environment for an extended period and re‐emerge when conditions are favourable (Raymond et al., 2010). Animal‐to‐human transmission was found to be via direct contact with animals and/or contaminated materials (slaughtering and skinning of ill or dead animals) (Blackburn et al., 2021; Maukayeva et al., 2019). In many developing countries, including Kazakhstan, animal husbandry is an essential sector in the livelihood of the majority of rural people. In rural areas, livestock is the primary source of income and is mainly kept for milk and meat production. The commercial smallholding dairy and beef farms are mostly market‐oriented and located around urban areas practising intensive management (Bayantassova et al., 2023; Issimov et al., 2022; Orynbayev et al., 2021).

Globally, approximately 20,000–100,000 human anthrax cases occur annually (Dutta et al., 2011). In Kazakhstan, anthrax is considered an enzootic disease (Shevtsov et al., 2021), primarily affecting animals; however, occasional human cases have been reported in West Kazakhstan, South Kazakhstan and Almaty regions (Ashford et al., 2004; Maukayeva et al., 2019; Shevtsov et al., 2021). Kazakhstan was formerly a part of the Union of Soviet Socialist Republics (USSR), and anthrax was first noted in the early 20th century (Abdrakhmanov et al., 2017). Throughout the 20th century, animal carcasses were interred on‐site or nearby until 1951 when a USSR Ministry of Agriculture decree mandated their incineration to curb soil contamination. Despite this, notable anthrax cases persisted in both animals and humans from the mid‐1950s to the 1980s. The long‐lasting survival of *B. anthracis* spores at these sites contributed to regional epidemic instability (Ashford et al., 2004). Moreover, land development led to increased livestock and human populations, resulting in heightened incidence in the 1950s. Although anthrax incidents decreased after 1969, the situation remained tense with over 50 outbreaks yearly until the early 1980s. A follow‐up animal mass vaccination campaign in 1981 and rigorous veterinary surveillance brought stability, maintaining an average of 20 animal outbreaks annually (Abdrakhmanov et al., 2017).

Between 2019 and 2023, 64 animal anthrax cases occurred in the livestock sector throughout the country (Office International des Epizooties [OIE], 2023). In developing countries, such as Kazakhstan, anthrax remains an occupational hazard of pastoralists and individuals engaged in close contact with animals or the processing of animal‐derived products (Ashford et al., 2004). According to Maukayeva et al. (2019), a total of 21 human anthrax cases were reported across five regions – Karaganda, Almaty, Pavlodar, East Kazakhstan and South Kazakhstan – between 2016 and 2018. Of these cases, four patients died due to generalized anthrax. Moreover, in June 2023, three human anthrax cases were registered in Zhambyl region among farmers who were involved in slaughtering infected animals (Suleymanova, 2023). It is believed that anthrax outbreaks in agrarian communities are mainly associated with consumption of undercooked meat, natural agents (movement of soil containing spores by wind, rain or flooding) and poor public education (Carlson et al., 2019; Hugh‐Jones et al., 2007). This in turn could define the knowledge, attitude and practices (KAP) of a given community regarding anthrax. Defining KAP towards anthrax within the community is pivotal to comprehensively assessing the level of public awareness and providing direction for efficient preventive and control strategies. To the best of our knowledge, no KAP studies regarding anthrax have been previously conducted in Kazakhstan. Therefore, this study was conducted with the aim of assessing KAP regarding anthrax among smallholder farms in selected districts in West Kazakhstan.

## 2 MATERIALS AND METHODS

### 2.1 Study area

**FIGURE 1:**
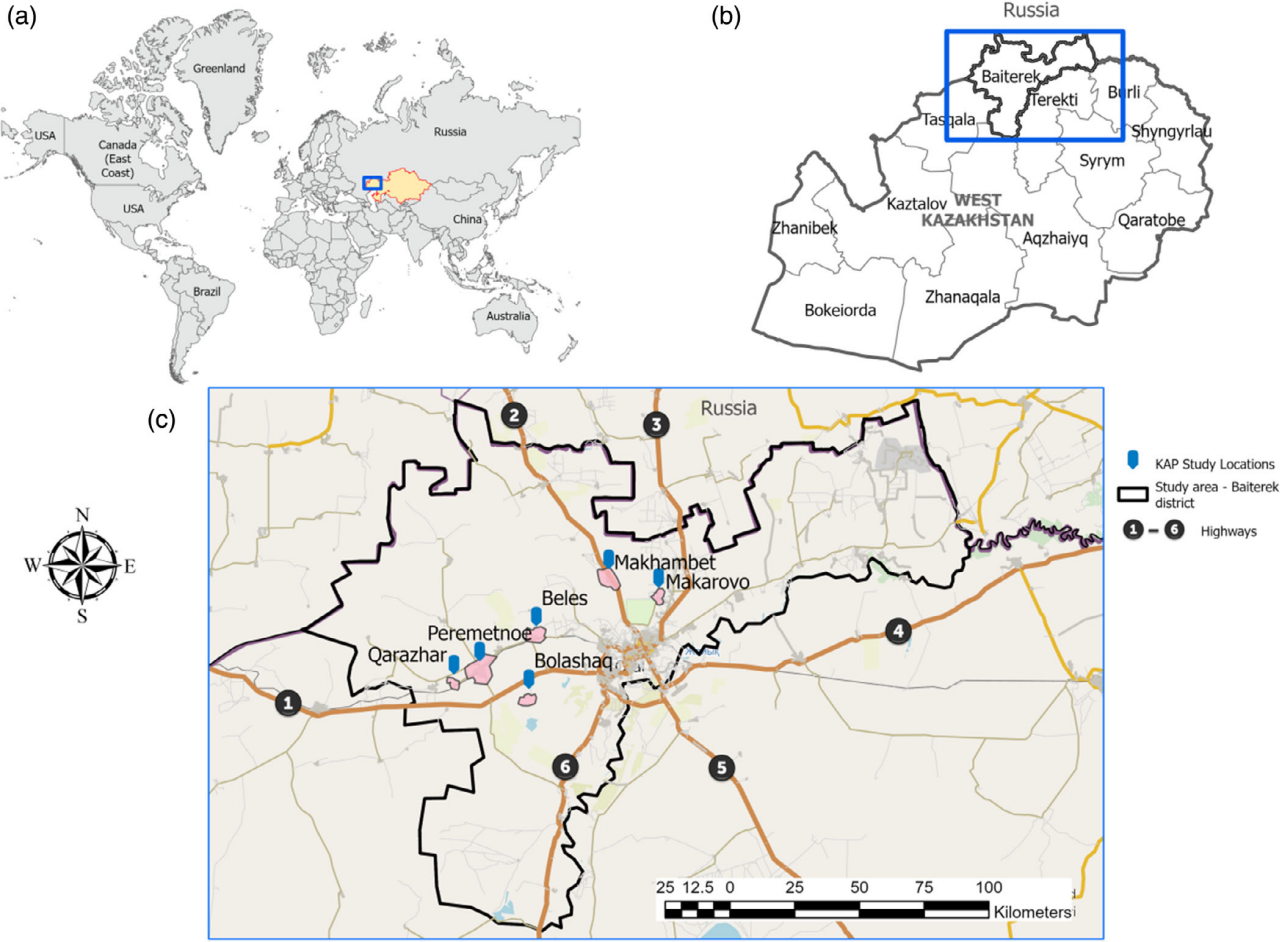
World map (a), region map (b) and study area map (c). Blue arrowheads represent knowledge, attitude and practice (KAP) study locations within Baiterek province.

The study area was located in the west part of Kazakhstan (51°38′–55°57′ N, 46°9′–46°4′ E) – West Kazakhstan region (Figure 1). The entire study area borders with the Russian Federation. Highways passing through districts investigated are depicted as numbers from 1 to 6: 1. Oral–Saratov (A298), 2. Oral–Samara (E121), 3. Oral–Bugulma (P246), 4. Oral–Orenburg (A305), 5. Oral–Aktobe (E38), 6. Oral–Atyrau (A28). The map was developed using ArcGIS Pro 2.8 (ESRI). The coordinate system used was WGS 1984 Web Mercator (Auxiliary Sphere).

### 2.2 Study design and study population

According to the national census, the population of the West Kazakhstan region is about 3 million people in an area of 736,241 km^2^. The livestock population in the same region is about 2.17 million head (GOV.KZ, 2023). Most of the farms practised a sedentary lifestyle. People who raised livestock were the participant of this study.

This study was conducted in West Kazakhstan, where a cattle anthrax outbreak was registered in 2009 (Shevtsov et al., 2020). A questionnaire‐based cross‐sectional study was conducted between January and May 2022 in Baiterek province. For this study, six districts were selected, namely Bolashak, Peremetnoe, Karazhar, Beles, Makhambet and Makarovo (Figure 1). These districts were purposively selected due to the high concentration of livestock and the history of frequent human anthrax cases (Shevtsov et al., 2021). Moreover, the West Kazakhstan region has several highways (Oral–Samara, Oral–Saratov, Aktobe–Orenburg, Aktobe–Orsk, Atyrau–Astrakhan) passing through these districts, connecting the region with the Russian Federation. These highways are also used for cattle import and export from or into the Russian Federation. Furthermore, there is a major highway (West China–West Europe or ‘The Belt and Road Initiative’ which connects the biggest agglomerations in the south part of Kazakhstan, including Almaty and Shymkent, to the western provinces of the country.

A comprehensive questionnaire containing 16 questions grouped into 2 main sections was used to collect the required information. The first section contained questions related to respondent and socio‐demographic information (age, sex, education status, occupation, animal ownership and location). The second section contained questions related to the knowledge, attitudes and perception of anthrax and its prevention methods.

The questionnaire was pre‐tested and modified after several interviews to remove ambiguity. The population data of the study districts were acquired from the administrative bodies of each respective district. The selection of participants was conducted through the application of systematic random sampling. Herd owners were interviewed using local Kazakh and Russian languages, depending on the participants’ preferences. Farmers were interviewed after explaining the aims of the study and receiving verbal consent. When the selected farm owner declined to participate in the study, another farm owner was selected to replace them. Interviews were conducted on the farm site, and participants were informed that the information provided will be kept confidential and utilized for research purposes only.

### 2.3 Sample size determination

The sample size was determined by considering an expected prevalence of 50%, a confidence level of 95% and a required precision of 5%. The method described by Thrusfield (2018) was used to generate sufficient data on the KAPs regarding anthrax among smallholder farmers in Baiterek province. Accordingly, the total number of participants required for the study was 330. However, for consideration of clustering and non‐response rates, approximately 48% of the sampling units were added, which gives a total sample size of 489 participants. Information on the occurrence of confirmed anthrax outbreaks and vaccination status in the selected districts between June and July 2009 was obtained from the District Veterinary Offices.

### 2.4 Data analysis

The collected data were entered into a Microsoft Excel spreadsheet, edited and analysed using the Python programming (version 3.9.7, Jupyter Notebook). To assess the understanding of respondents regarding anthrax, including its symptoms, transmission and control/prevention methods, frequency distribution tables were employed. These tables quantified the knowledge based on socio‐demographic factors, such as age, sex, educational level, district, occupation and animal ownership. Furthermore, a logistic regression model was utilized, along with 95% confidence intervals, to examine the relationship between the outcome variable (e.g. knowledge about the disease's zoonotic nature) and the aforementioned socio‐demographic variables. A value of *p* ≤ 0.05 was designated as statistically significant.

## 3 RESULTS

### 3.1 Socio‐demographic characteristics

The socio‐demographic characteristics of the participants in the study area are shown in Table 1. The participation rate was 88%. The mean age of participants was 49 years (range, 25–75 years). Males constituted the majority of participants (73%). About one third (36%) of participants had at least tertiary education, whereas approximately a quarter (22%) had a low literacy level. The occupation of participants was mainly agriculture‐oriented (91%). The remaining 8% of participants were involved in other businesses, such as running grocery stores, which indicates that farming was not their primary source of income. However, these individuals still maintained a small number of cattle (1–2 heads, mostly 1 head) for milking. In the area studied, cattle rearing was a predominant occupation among two thirds (67%) of farmers, followed by sheep (13%), goats (9%) and horses (9%).

**TABLE 1:** Socio‐demographic characteristics of participants (*n* = 489).

Variables	Number of participants	Frequency % (95% CI)
Districts		
Beles	92	18.56 (17.5–20.1)
Bolashak	87	17.67 (16.2–19.4)
Peremetnoe	85	17.45 (16.7–19.2)
Makhambet	84	17.43 (15.8–19.2)
Karazhar	72	14.15 (12.0–16.0)
Makarovo	69	14.74 (12.7–15.5)
Age		
45–54	247	50.4 (48.4–51)
35–44	104	21.7 (20.0–21.7)
55–64	86	17.15 (15.8–18.5)
25–34	31	6 (5.0–8.0)
65–74	21	4.75 (1.9–6.0)
Sex		
Male	360	74 (68.8–77.1)
Female	129	26 (24.2–29.5)
Education		
Secondary	200	40.6 (39.1–42.2)
Tertiary	180	36.6 (35.3–39.0)
Primary	109	22.8 (19.0–23.6)
Occupation		
Agriculture	446	91.56 (88.6–94.1)
Business	43	8.44 (6.6–10.3)
Livestock species		
Cattle	331	67.7 (65.4–68.6)
Sheep	65	13.34 (12.0–14.7)
Goat	47	9.67 (8.2–11.4)
Horses	46	9.29 (7.0–10.8)

### 3.2 Knowledge of respondents regarding anthrax

Knowledge of respondents towards anthrax is shown in Table 2. All participants interviewed had heard about the existence of anthrax as a disease. Of the total 489 interviewees, 53% of respondents reported that they acquired information about anthrax from friends, 26% from neighbours, 11% from veterinary health practitioners and 8% from media, respectively. Overall, 17% of participants were able to describe at least some animal anthrax symptoms, whereas 12% were able to describe some human anthrax symptoms. Overall, 36% of participants were certain about the zoonotic nature of the disease and were aware of the potential of pathogen transmission from animal to human via eating, whereas 1% and 11% of them knew the possible mode of transmission of anthrax via contaminated soil and direct contact with infected meat. The remaining 51% of respondents demonstrated a lack of knowledge on the transmission mode of anthrax from animal to human. About one third of participants (35%) were certain that vaccination is an effective method in anthrax prophylaxis (Table 3).

**TABLE 2:** Knowledge of the participants towards anthrax (*n* = 489).

Variables	Number of participants	Frequency % (95% CI)
Source of information about anthrax		
Friends	263	53.43 (51.0–54.0)
Neighbours	127	26.25 (25.7–27.5)
Veterinary health practitioners	56	11.67 (9.6–12.5)
Media	43	8.65 (7.7–10.1)
Knowledge about symptoms of animal anthrax		
No	405	82.3 (79.3–84.1)
Yes	84	17.7 (16.8–19.0)
Knowledge about symptoms of human anthrax		
No	430	87.6 (86.7–88.2)
Yes	59	12.4 (11.3–12.9)
Knowledge about transmission route of anthrax from animal to human		
Do not know	252	51.1 (47.2–55.5)
Eating	176	36.6 (34.8–38.1)
Infected meat	57	11.2 (9.9–12.1)
Soil	4	1.1 (0.3–2.0)
Preventive method of anthrax (vaccination)		
No	316	64.4 (63.8–66.2)
Yes	173	35.6 (34.4–37.1)

**TABLE 3:** Attitude and practices of participants towards anthrax (*n* = 489).

Variables	Number of participants	Frequency % (95% CI)
Where do you get your anthrax vaccine (source of vaccine)		
State veterinary service	427	87.2 (85.4–89.0)
Other	62	12.8 (11.4–13.9)
Do you vaccinate your animals against anthrax		
Yes	344	70 (69.9–71.3)
No	145	30 (28.2–33.0)
Respondents considered anthrax a fatal disease		
Yes	299	61 (60.6–62.2)
No	137	28 (27.4–29.2)
Do not know	53	11 (9.0–12.0)
Participation in affected animal slaughtering		
Yes	4	99 (97.7–100.4)
No	485	1 (0.5–1.1)

### 3.3 Attitude and practices of participants regarding anthrax

Mass vaccination is considered the most effective control measure in restraining infectious diseases. In Peremetnoe district, the vaccination status of livestock was the highest, accounting for 91.2%, followed by Bolashak (84.4%), Beles (82%), Makhambet (77%), Karazhar (76%) and Makarovo (64%). An overwhelming majority of participants (87%) declared that the anthrax vaccine was provided by the State veterinary service and local veterinary health practitioners administered the vaccine. Meanwhile, 12% of respondents reported obtaining the vaccine from another source. Most of the respondents (70%) were interested in vaccinating their livestock against anthrax. Respondents in the age group of 45–54 years demonstrated significantly higher vaccination rates for their animals (45% [95% CI: 38.4–52.0]; *p* < 0.025) than those 25–34 (23% [95% CI: 19.2–25.4]; *p *< 0.12) and 65–74 (17% [95% CI: 12.6–20.1]; *p* < 0.093) years old. Respondents from Beles district (20% [95% CI: 17.1–24.7]; *p* < 0.005) had a significantly higher level of awareness regarding anthrax mortality than respondents from Bolashak (9% [95% CI: 7.1–12.4]; *p* < 0.104). Nearly two thirds of respondents (61%) believed that anthrax is a lethal disease. The vast majority of participants (99%) declared that they had not been involved in slaughtering diseased animals.

## 4 DISCUSSION

Anthrax (*B. anthracis*), a Gram‐positive bacterium capable of forming spores, presents a significant global public health concern (Dutta et al., 2021). It is prioritized as the second most important zoonotic disease agent in developing nations, with approximately 63.8 million impoverished smallholder farmers and 1.1 billion livestock residing within anthrax‐endemic areas worldwide (Carlson et al., 2019). Being classified as a neglected zoonotic disease, it inflicts substantial economic losses on the livestock sector through productivity decline and sudden animal death (Nayak et al., 2019; Ngetich, 2019).

Endemic in most Central Asian regions, anthrax frequently affects rural areas of Kazakhstan (Maukayeva et al., 2019; Sweeney et al., 2011), impacting humans and livestock across Africa and Asia (Kutmanova et al., 2022; Tyulegenov et al., 2022). Human behaviour plays a crucial role in anthrax epidemiology (Dutta et al., 2021). Globally, in agricultural sector and industrial activities, human infection is mainly associated with consuming infected meat and close contact with affected animals or contaminated materials (Blackburn et al., 2021; Issimov et al., 2022; Mwakapeje et al., 2018; Seisenov et al., 2023). Additionally, socio‐cultural practices that involve slaughtering and underreporting of sick animals, consuming or handling meat from diseased animals, and improper disposal of dead carcasses have been associated with anthrax dissemination in developing countries (Islam et al., 2024; Zorigt et al., 2021).

Anthrax outbreaks among livestock in Kazakhstan have been recorded since the early 1930s (Abdrakhmanov et al., 2017). Since then, until 2023, 4128 anthrax outbreaks have been reported (Abdrakhmanov et al., 2017; OIE, 2023). In the Baiterek outbreak, nine strains were discovered at the animals’ slaughtering site and were subsequently identified as *B. anthracis* (Shevtsov et al., 2021; Shevtsov et al., 2020). In the same outbreak, anthrax strains were also isolated from humans; however, all information regarding human cases was anonymized. Initially, this KAP study was planned to be carried out in Borli and Baiterek provinces of West Kazakhstan oblast, as these districts were reported to the World Organization for Animal Health as anthrax positive in 2009 (Shevtsov et al., 2020). However, most residents from the Borli province refused to participate in this study as they were afraid of the stigma that could negatively affect their farming business. For this reason, the number of districts surveyed in Baiterek province was increased from an initial three to six districts to collect sufficient data.

Large‐scale vaccination of livestock is the most efficient tool to halt and control future anthrax emergence (Kasradze et al., 2018; Rao et al., 2019; Zhugunissov et al., 2023). In Kazakhstan, the anthrax vaccine is provided by State veterinary service and is believed to meet the demand of the livestock sector. In our study, the highest vaccination rate of livestock was observed in Peremetnoe (91%) and the lowest in Makarova (64%). From personal communication, however, it was revealed that despite the lack of vaccine deficiency, 29% of study participants prefer not to vaccinate their livestock against anthrax. They stated that the livestock anthrax vaccine might not be safe enough, and they can acquire the infection when consuming animal products following vaccination. Respondents from Beles district aged 45 to 54 displayed higher animal vaccination rates (45%) compared to those aged 25–34 and 65–74. This could be explained by a specific age group's proactive approach towards vaccination, possibly due to their greater experience or awareness regarding the disease and the benefits of vaccination. Another possible explanation could be improved access to veterinary services, potentially due to proximity to the administrative centre, Uralsk.

In the current study, nearly a quarter (22%) of participants were literate at the primary school level. In turn, this may lead livestock farmers with poor literacy to hide the truth about anthrax suspicious cases to the local authority or hide their involvement in slaughtering sick animals and subsequent selling and consuming infected meat.

According to the data, 51% farmers showed a lack of knowledge of anthrax transmission. Respondents aged 55–64 demonstrated higher knowledge (28%) about symptoms of animal anthrax among age groups surveyed. Among these respondents, more males (59%) than females (41%) were aware of the fatality of anthrax. Overall, 71% of these farmers interviewed were entirely reliant on cattle rearing. Moreover, among all participants surveyed, about 80% reported that they heard about anthrax from friends and neighbours. Such a source of information, in turn, increases the probability of misconceptions and myths regarding the disease (Sitali et al., 2017). Of the study population, only 12% and 17% of participants could describe some symptoms peculiar to human and animal anthrax.

This retrospective study has several limitations that need to be acknowledged. First, the most important constraint was the reliability of data obtained from respondents. Farmers could underreport incident cases and conceal their losses due to the stigma associated with anthrax. Second, the farmers interviewed remembered events differently; thus, it was not feasible to establish causation due to recall bias.

It is suggested that in developing countries like Kazakhstan, the government must develop strategies by launching training programmes to improve the public health awareness and preparedness of farmers and veterinary personnel to withstand future outbreaks, including anthrax. Practically, this could be achieved by distributing leaflets containing relevant epidemiological information of the disease: prophylaxis and treatment methods, early diagnosis, mode of transmission and information on anthrax burial sites. Leaflets with images and simplified text have the advantage of conveying information through visual aids, which can benefit both illiterate and literate individuals. Visual representations and simple graphics can effectively convey key messages, ensuring that important information reaches all members of the community, regardless of their literacy level. Similarly, utilizing interactive training sessions that engage participants through demonstrations and discussions can be effective to ameliorate public health awareness among farmers.

Furthermore, social media messengers such as ‘WhatsApp’ and/or Facebook which are increasingly becoming popular among the rural population in Kazakhstan, can also be used for this purpose. All these combined will inevitably contribute to promoting the biosecurity consciousness of the population and navigating the control and prevention of public health threat diseases in Kazakhstan.

## 5 CONCLUSION

To the best of our knowledge, this is the first KAP study conducted among livestock farmers involving anthrax in Kazakhstan. The outcomes obtained in this study demonstrate that all smallholder farmers had a paucity of knowledge about anthrax; as such, most of them failed to describe its symptoms. Over half of the study population was unaware of anthrax transmission. The study also revealed that the majority of the community members did not receive consistent, adequate and continuous information regarding the disease from public health and veterinary professionals. Given that there is a significant density of livestock population in the West Kazakhstan oblast, the data obtained will enhance insight into anthrax epidemiology and guide the development of prevention strategies suitable for use in the area investigated.

## AUTHOR CONTRIBUTIONS

**Altyn Kulpiisova**; **Nadezhda Burambayeva**; and **Arman Issimov**: Investigation; methodology; supervision; writing–original draft. **Zukhra Aitpayeva**; **Lyailya Ussenova**; **Assel Paritova**; **Assylbek Zhanabayev**; **Temirlan Bakishev**; **Spandiyar Tursunkulov**; **Tileubek Kitapbay**; **Aspen Abutalip**; **Assiya Mussayeva**; **Yerzhan Ospanov**; **Urzhan Omarbekova**; **Bauyrzhan Turalin**; **Vladislav Sapa**; **Marat Aisin**; **Altyn Kulpiisova**; **Gulnara Baikadamova**; **Salbak Chylbak‐ool**; **Elena Pakhomova**; and **Nurkuisa Rametov**: Conceptualization; data curation; formal analysis; funding acquisition; software.

## CONFLICT OF INTEREST STATEMENT

The authors declare that the research was conducted in the absence of any commercial or financial relationships that could be construed as a potential conflicts of interest.

### ETHICS STATEMENT

The protocol was approved by the Human Ethics Committee of the K. Zhubanov Aktobe Regional University (permit number: 01‐05‐07‐21‐2020). The purpose and methods of the current study were thoroughly explained to all participants, and informed oral consent was obtained and documented in the questionnaire.

### PEER REVIEW

The peer review history for this article is available at https://publons.com/publon/10.1002/vms3.1553.

## Data Availability

The datasets containing all data analysed and supporting the results of this study will be available upon request from the corresponding author.
